# Exploring the Mechanism of Action of Canmei Formula Against Colorectal Adenoma Through Multi-Omics Technique

**DOI:** 10.3389/fcell.2021.778826

**Published:** 2021-11-29

**Authors:** Cui Guo, Xiaoqiang Liu, Yimin Xu, Xinyue Han, Runnan Xie, Xiangxue Meng, Yuan Li, Tongyu Chen, Zhihong Cheng, Xiaoling Fu

**Affiliations:** ^1^ Second Department of Oncology, Yueyang Hospital of Integrated Traditional Chinese and Western Medicine, Shanghai University of Traditional Chinese Medicine, Shanghai, China; ^2^ Graduate School of Liaoning University of traditional Chinese Medicine, Shenyang, China; ^3^ Department of Pain, Shibei Hospital, Shanghai, China; ^4^ Miaohang Town Community Health Service Center, Shanghai, China; ^5^ Cardiothoracic Surgery Department, Yueyang Hospital of Integrated Traditional Chinese and Western Medicine, Shanghai University of Traditional Chinese Medicine, Shanghai, China; ^6^ China State Institute of Pharmaceutical Industry, National Pharmaceutical Engineering Research Center, Shanghai, China

**Keywords:** multi-omics, colorectal adenoma, Canmei formula, intestinal flora, LHPP

## Abstract

**Background:** Canmei formula (CMF) is a traditional Chinese medicine compound with definite effect on the prevention and treatment of colorectal adenoma (CRA). CMF can prevent the transformation of intestinal inflammation to cancer. This study explored the mechanism of action of CMF in anti-CRA using multi-omics techniques.

**Method:** The mice were randomly divided into four groups: blank group (Control), high-fat diet (HFD) + AOM/DSS colorectal adenoma model (ADH) groups, Canmei formula treatment group (ADH-CMF) and sulfasalazine treatment group (Sul). Except for the blank group, ADH model was established in the other three groups by intraperitoneal injection with AOM reagent, and then mice were given 2.5% DSS in free drinking water and high-fat diet. The mice in the blank group and ADH groups were intragastrically perfused with normal saline, and the mice in the other two groups were treated with corresponding drugs for 20 weeks. During this period, the changes of physical signs of mice in each group were observed. The differentially expressed genes and proteins in the Control group, ADH group and ADH-CMF group were detected by RNA-seq transcriptome sequencing and Tandem Mass Tags (TMT) quantitative proteomics. After the combined analysis and verification, the key targets were analyzed by gene ontology (GO) and Kyoto Encyclopedia of Genes and Genomes (KEGG). Moreover, the changes of intestinal flora in mice of the three groups were examined.

**Results:** A total of 2,548 differential genes were obtained by transcriptomics analysis, and 45 differential proteins were obtained by proteomics analysis. The results of proteomics data and experimental verification showed that CMF mainly affected the Phospholysine Phosphohistidine Inorganic Pyrophosphate Phosphatase (LHPP) target. GO analysis showed that the targets of CMF were involved in the biological processes such as cellular process, metabolic process and biological regulation. KEGG analysis showed that those genes were involved in oxidative phosphorylation, cell senescence, and metabolic pathways. Studies have shown that LHPP overexpression impeded colorectal cancer cell growth and proliferation *in vitro*, and was associated with a change in PI3K/AKT activity. The results of 16S DNA high-throughput sequencing showed that CMF could effectively regulate the abundance of Bifidobacterium, Candidatus_Saccharimonas and Erysipelatoclostridium in the intestinal flora at the genus level.

**Conclusion:** CMF regulates LHPP via the PI3K/AKT signaling pathway. CMF affects the abundance of specific intestinal flora and can regulate the disorder of intestinal flora to achieve the role of prevention and treatment of CRA.

## Introduction

Colorectal cancer (CRC) is one of the digestive tract malignant tumors that seriously endangers human health. CRC is also the second leading cause of cancer-related death worldwide (Chen et al., 2020). Colorectal adenoma (CRA) is a common precancerous lesion. More than 90% of CRC is caused by CRA carcinogenesis (Zhang et al., 2020), which is characterized by the increased release of inflammatory mediators in the aberrant crypt foci, and proliferation of mucosal epithelial cells in local intestinal tissues (Geng et al., 2018). Detection and removal of CRC in the early or adenoma stage is generally considered to be the most effective way to reduce CRC-related mortality (Bray et al., 2017). Therefore, it is extremely urgent to elucidate the underlying molecular mechanisms of CRC and develop new therapeutic strategies.

Traditional Chinese medicine plays an important role in the prevention and treatment of diseases. Through clustering and frequency analysis and data mining, our research group found that the core drug for the treatment of CRA was CMF, which consists of Mume Sieb 30 g and Marci Hieronymi 30 g (Zhang et al., 2019). In addition, we successfully established a CRA mouse model induced by high-fat diet in combination with AOM/DSS treatment (Guo et al., 2021). However, at present, the study on CMF remains at the level of overall clinical efficacy, and the mechanism of its action has not been reported, which greatly hindered its promotion and application in clinical practice.

The occurrence and development of intestinal adenoma is affected by environment and internal genes. In terms of environment, intestinal flora plays an important role (Singh et al., 2017). The imbalance of intestinal flora leads to the increase of pathogenic bacteria and the destruction of intestinal mucosa, resulting in DNA damage and inflammatory reaction, leading to carcinogenesis (Kawanishi et al., 2017). Therefore, the regulation of intestinal flora plays important roles in the prevention and treatment of intestinal adenoma (Zhang Y. et al., 2021). Traditional Chinese medicine is usually administered orally, and interacts with intestinal flora through the digestive tracts (Guo et al., 2021). Studies have shown that Traditional Chinese medicine exerts therapeutic effects by regulating intestinal flora structure, increasing beneficial bacteria and reducing pathogenic bacteria (Zhang H.-Y. et al., 2021).

Understanding the material and mechanism of action of traditional Chinese medicine compound prescription is the key to realize the modernization of traditional Chinese medicine (Li and Weng, 2017; Zhou et al., 2017). It is an inevitable trend to apply new technologies and new means of modern scientific techniques to study the complex system of traditional Chinese medicinal prescriptions (Wang et al., 2018). In the post-gene era, proteomics and transcriptome have become new technologies and are the focus of system biology research, the correlation analysis between the two can provide a comprehensive understanding of gene expression, and through mutual supplement and comparison, more complete expression information of organisms can be obtained, which can be applied to the research of traditional Chinese medicine (Manzoni et al., 2018; Paczesny, 2018).

This study investigated the mechanism of action of CMF on CRA from the perspective of gene, protein expression and intestinal flora through proteomics and transcriptomics technology. It will provide a rational clinical application of CMF, and new ideas and methods for the treatment of CRA.

## Materials and Methods

### Drugs and Reagents

Marci Hieronymi (Anhui, batch number 2018111608), purchased from Shanghai Huapu traditional Chinese Medicine Co., Ltd., was identified as larvae of the fourth ∼ fifth instar of *Bombyx mori* Linnaeus infected (or artificially inoculated) *Beauveria bassiana* (Bals). Mume Sieb (Sichuan, batch number 18100901), purchased from Shanghai Dedatang traditional Chinese Medicine Co., Ltd., Mume Sieb was identified as Prunus mume Sieb. et Zucc. Near-ripe fruits were dried.

### Drug Preparation and Component Analysis by Mass Spectrometry

The 500 g of Mume Sieb and 500 g of Marci Hieronymi were mixed with honey bran according to the ratio of Mume Sieb and Marci Hieronymi at 1:1, soaked 10 times in distilled water for 2 h, refluxed and extracted for 1 h, then 8 times of distilled water was added for reflux and extraction for 1 h. The mixture was filtered through three layers of gauze and the filtrate was combined twice. Organic acids were separated by pH method, polypeptides were separated by macroporous resin, 90% ethanol was added and extracted by rotary evaporator and concentrated under reduced pressure. Finally, the effective components of CMF extracted by water and alcohol precipitation were used to treat mice in the CMF group. In addition, 100 mg of sulfasalazine powder was added with 1% CMC murine Na 20 ml and pure water to a fixed volume of 40 ml in the 50 ml centrifuge tube, with the concentration of 2.6 mg/ml. All the drugs prepared above were stored in a refrigerator at 4°C and shaken well before intragastric administration. The above drug extraction process was completed by the Pharmaceutical Research Room of Yueyang Hospital affiliated to Shanghai University of traditional Chinese Medicine (Zhang et al., 2019).

To identify the components of Mume Sieb and Marci Hieronymi extract, Q Exactive high-performance benchtop quadrupole-Orbitrap LC-MS/MS was performed. The main components in the extract of CMF were identified according to the elemental composition data determined from accurate mass measurements and comparison with the literature data (this step was completed by our research group in the previous study) (Zhang et al., 2019).

### Animal Grouping and Animal Modeling

Thirty-two SPF C57BL/6 mice, male, 7–8 weeks of age, weighing 17–20 g, were purchased from Shanghai Jihui Experimental Animal Feeding Co., Ltd (Certificate No: 20170012005900). The mice were maintained in the animal facility with constant temperature 23 ± 2°C, constant humidity 50 ± 10%, and 12/12 h light/dark cycle. This experiment was approved by the Experimental Animal Ethics Committee of Shanghai University of Traditional Chinese Medicine (ethics number: YYLac-2019-042-1).

The mice were intraperitoneally injected with AOM (12.5 mg/kg) (Ameresco)reagent on the first day of the experiment. On the sixth, 27th and 46th day of the experiment, mice were administered 2.5% DSS (sigma) drinking water for 5 days. Mice were given routine aqueous solution provided by the laboratory at other times. At the same time, they were fed with a high-fat diet to establish the AOM/DSS + HFD -induced intestinal adenoma (ADH) animal model. The normal diet consisted of a standard laboratory chow with 5% fat, whereas the HFD contained 45% fat (this step was completed by the research group in the previous study) (Guo et al., 2021).

Mice were randomly divided into four groups with eight mice in each group. The grouping was as follows: Control: normal saline was given intragastrically (0.4 ml/mice, once a day); ADH: Gavage with normal saline (0.4 ml/mice, once a day); ADH-CMF: CMF water extract and alcohol precipitation component suspension was used for gavage (658 mg/kg, 0.4 ml/mice, once a day); Sul: Salazosulfadiazine was given orally (60 mg/kg, 0.4 ml/mice, once a day). Note: the drug dose was based on Experimental Zoology: mouse dose (mg/kg) = human dose 65 kg 12.33 times; the dose of CMF water extraction and alcohol precipitation group was converted by the actual drug extraction rate (18%). During the experiment, the food and drinking water was given freely, the cushion was changed regularly, and the body weight was weighed once a week. During the period of drinking DSS aqueous solution, the nutritional status, hair, appetite, activity state, stool shape and whether there was occult blood or visible hematochezia were observed every day. According to the literature, after the occurrence of adenoma at the 12th week, drug intervention treatment was given by intragastric administration of 0.4 ml every afternoon for 20 weeks. During this period, the changes of physical signs of mice in each group were observed, and the body weight of mice was recorded. At the end of treatment, colon tissues were taken. The changes of colon length, the occurrence of colorectal adenoma and HE histopathological staining were observed.

### Sequencing and Analysis of Differentially Expressed Genes

Eukaryotic mRNA sequencing is performed using the IlluminaNovaseq6000 sequencing platform. The Illumina TruseqTM RNA sample prep Kit is used to construct the library for the sequencing experiment. The quality of RNA sequencing data was controlled by FastQC software, and the known IlluminaTruSeq junction sequence. Low quality sequence and ribosomal RNA sequence were removed. The reserved sequence was mapped to the mouse reference genome by Hisat2, each gene count in the reserved sequence was screened by Stringtie. The gene count was normalized by TMM, and the FPKM was calculated by Perl script. The difference of gene expression between the model group and the control group, the model group and the CMF group was analyzed by EdgeR. *p* value <0.05 and fold difference >1.2 were used as significant difference criteria.

### Quantitative Proteomics was Performed by Multiplexed Tandem Mass Tag Ms and Differentially Expressed Proteins Analysis

The proteins were extracted from intestinal tissues, digested with trypsin, and labelled with TMT reagents. The pooled peptides were separated into 15 fractions using a C18 column (Waters BEH C18 4.6 × 250 mm, 5 µm) on a Rigol L3000 HPLC. When the protein abundance ratio is 1.2 times or more, and *p* < 0.05, the protein was considered as differential protein. Venny diagram was used to screen highly expressed differentially expressed proteins (Control group vs. ADH group and ADH group vs ADH-CMF group) and low expression of differentially expressed proteins (Control group vs. ADH group and ADH group vs. ADH-CMF group).

### Association Analysis

With the above results, the Shengxin alignment calculation was used to achieve efficient and personalized data mining purposes, with fold difference in gene expression ≥2, p-value ≤ 0.001, fold difference in protein expression ≥1.5, p-value ≤ 0.05 as standards. Differentially expressed genes and proteins were screened for association analysis and Person correlation coefficients were calculated. Further data mining was performed on key molecules from the protein and RNA levels to reveal the relevant mechanisms.

### Bioinformatics Prediction

The David (https://david.ncifcrf.gov/) to GO online and KEGG analysis were used to compare the genes identified between different samples. The selected targets were analyzed using the GEPIA database (http://gepia.cancer-pku.cn/). The present study used gene data from The Cancer Genome Atlas (http://gepia2.cancer-pku.cn/#index) in order to evaluate the differences in targets mRNA expression between CRC tissues and matched noncancerous tissues. The median mRNA expression of targets was regarded as the cut-off value to distinguish patients with high and low expression. The overall survival data were collected for further analysis.

### The Parallel Reaction Monitoring and qRT-PCR Verification of Differential Target Expression

The peaks of the original PRM data were extracted by Skyline, and the three and four ions with high abundance from Y3 to Yn-1 were selected for quantitative analysis, and checked and corrected manually. The peak area results of each target peptide after Skyline analysis were derived, including the target peptide sequence, the target protein name, and the quantitative peak area of each peptide. The sub-ion peak area of the peptide in the target protein was integrated and analyzed.

The RNeasy Lipid Tissue MiniKit kit was used to extract total RNA from intestinal tissues, and qPCR was performed following the manufacturer’s instructions.

### Analysis of Intestinal Microflora of Mice With Intestinal Adenoma Treated With CMF

Mice in each group were placed in autoclaved cushion-free cages to defecate freely, and their feces were quickly collected with sterilized tweezers in a sterilized 1.5 mLEP tube and stored in a refrigerator at −80°C for later use. The intestinal microflora of mice was quantitatively and qualitatively detected by 16SrRNA high-throughput sequencing technology, and the diversity and abundance of intestinal microflora of mice in model group and ADH-CMF group were analyzed. The fecal flora sequencing and bioinformatics were analyzed by Shanghai Jingzhou Gene Technology Co., Ltd. for.

### Data Statistics

SPSS 21 was used for statistical analysis. The actual data were expressed in the form of “mean ± SE” . Unpaired students’ t-test was used to compare the means of two groups. One-way analysis of variance and Adonis were used to compare the means of more than two groups. A level of *p* < 0.05 was considered statistically significant.

## Results

### Main Active Ingredients of CMF

After UPLC separation, each component of the sample was well separated. A total of 358 chemical components related to CMF were retrieved. Unifi software was used to check the compounds in the library and match 290 known compounds, 36 compounds were matched by ESI + mode, and 29 compounds were matched by ESI- mode. There were 15 compounds repeated by ESI + mode and ESI- mode, and a total of 50 compounds were obtained after deletion of the duplicates. Among them, folic acid and quercetin were the two compounds with the highest oral bioavailability and the best medicinal properties. The other ingredients with oral availability of greater than 50% were L-(-)-Tyrosine, malic acid, Quinic acid, Adenine, Glutamic acid, Anthranilic acid, Citric acid, Hypoxanthine, and D-Glucosamine et al (Supplementary Table S1).

### Therapeutic Effect of CMF on Intestinal Adenoma in Mice

**FIGURE 1:**
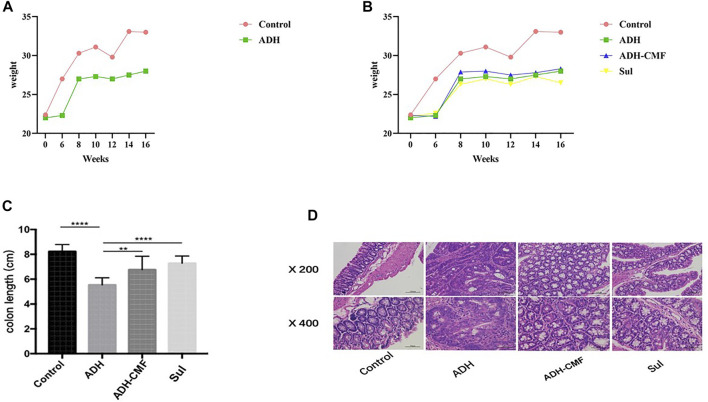
Effect of CMF on morphological changes of intestinal Adenoma in mice. **(A)** body weight of mice in Control group and ADH group; **(B)** Change of body weight of mice in each group. **(C)** Statistical analysis of colonic length in each group of mice; ****p < 0.001; **p < 0.01; **(D)** Results of HE staining in colonic tissues of each group.

CMF effectively reduced the size of adenomas in the range of 1–3 mm and >3 mm. Compared with ADH group, the number of adenomas in ADH-CMF group and Sul group is reduced, and the inhibitory effect is statistically different (Table1). Based on the statistical results, compared with the ADH group, the number and size of intestinal adenoma were reduced to varying degree after drug treatment, and the treatment effect was similar among the drug groups, with no statistical difference (Table 2). At the beginning of the experiment, there was no significant difference in body weight among the control group, ADH-CMF group, ADH group and Sul group (*p* > 0.5). After 6 weeks of modeling, compared with the control group, the body weight of ADH group mice decreased significantly. At the end of the third dose of DSS, the body weight of model mice reached the lowest. In the subsequent experiment, the body weight of mice tended to increase most likely due to tolerance (Figure 1A). The weight of mice in the control group remained steadily during the experiment. The weight change of mice in the ADH-CMF group treated with the CMF showed the same trend as that of ADH group. The weight of mice in the Sul group was the lowest (Figure 1B). Mice of the ADH-CMF group and ADH group were significantly different from that of Control group (*p* < 0.001). The macroscopic phenotypic results of the mice colon showed that the average length of the colon in the Control group was about 8 cm, the average length of the colon in the ADH group was about 5 cm. The colonic length of mice in ADH-CMF group and Sul group was significantly longer than that in model mice, indicating that CMF alleviated the colonic inflammation in mice (Figure 1C). Histologically, mice in Control group had normal glandular structure, clear mucosal layer structure, no mucosal ulcer, and no obvious atypia in glandular epithelial cells. In ADH group, colon histopathology showed that the glands were densely packed and arranged in a sieve-like structure, and the epithelial cells of the glandular cells were obviously dysplastic, and the nuclei were significantly enlarged and pathological nuclear mitosis was observed. Histological examination of the colon of mice in ADH-CMF group showed normal glandular structure and no obvious atypia in glandular epithelial cells. Histology of the colon of mice in Sul group showed normal glandular structure, glandular epithelial cells had no obvious atypia, and some plasma cells were visible (Figure 1D).

**TABLE 1:** The number of adenomas in the range of 1 ∼ 3 mm and larger than 3 mm in the intestinal tract of mice.

Group	Number of adenomas ( $\bar{x}\pm \text{s}$ )		Inhibition rating (%) (1~3 mm)
	1~3 mm	>3 mm	
Control	0.00	0.00	—
ADH	5.18 ± 1.60*#	1.95 ± 1.02*#	0.00
ADH-CMF	2.75 ± 1.04*	0.00*	46.91
Sul	4.44 ± 1.94#	0.00*	14.29

*p < 0.05 vs. ADH; #*p* > 0.05 vs. ADH; *#*p* < 0.05 vs. control.

**TABLE 2:** Adenoma size of colon (mm).

Group	Adenoma size ( $\bar{x}\pm \text{s}$ )
	Colon (mm)
Control	0.00
ADH	2.25 ± 1.9*
ADH-CMF	1.22 ± 1.29#
Sul	1.02 ± 0.85#

*p < 0.05 vs. Control; #*p* > 0.05 vs. ADH.

### Transcriptomic Results

#### Quality Assessment of Sequencing Data

In the sequencing library established in this experiment, the original data of nine samples are 4.8 × 10^7^–6.0 × 10^7^, with a total of 4.79 × 10^8^ reads. After filtering, about 4.75 × 10^8^ (99.16%) of the original data is retained as a high-quality reading segment (clean reads). The difference of reads of these nine samples is small. The percentage of bases with quality value ≥20 (Q20) was greater than 98.08%, and the percentage of bases with quality value ≥30 (Q30) was greater than 94.4%, indicating that the sequencing results are good and can be used for analysis (Supplementary Table S2).

#### Differential Gene Analysis

**FIGURE 2:**
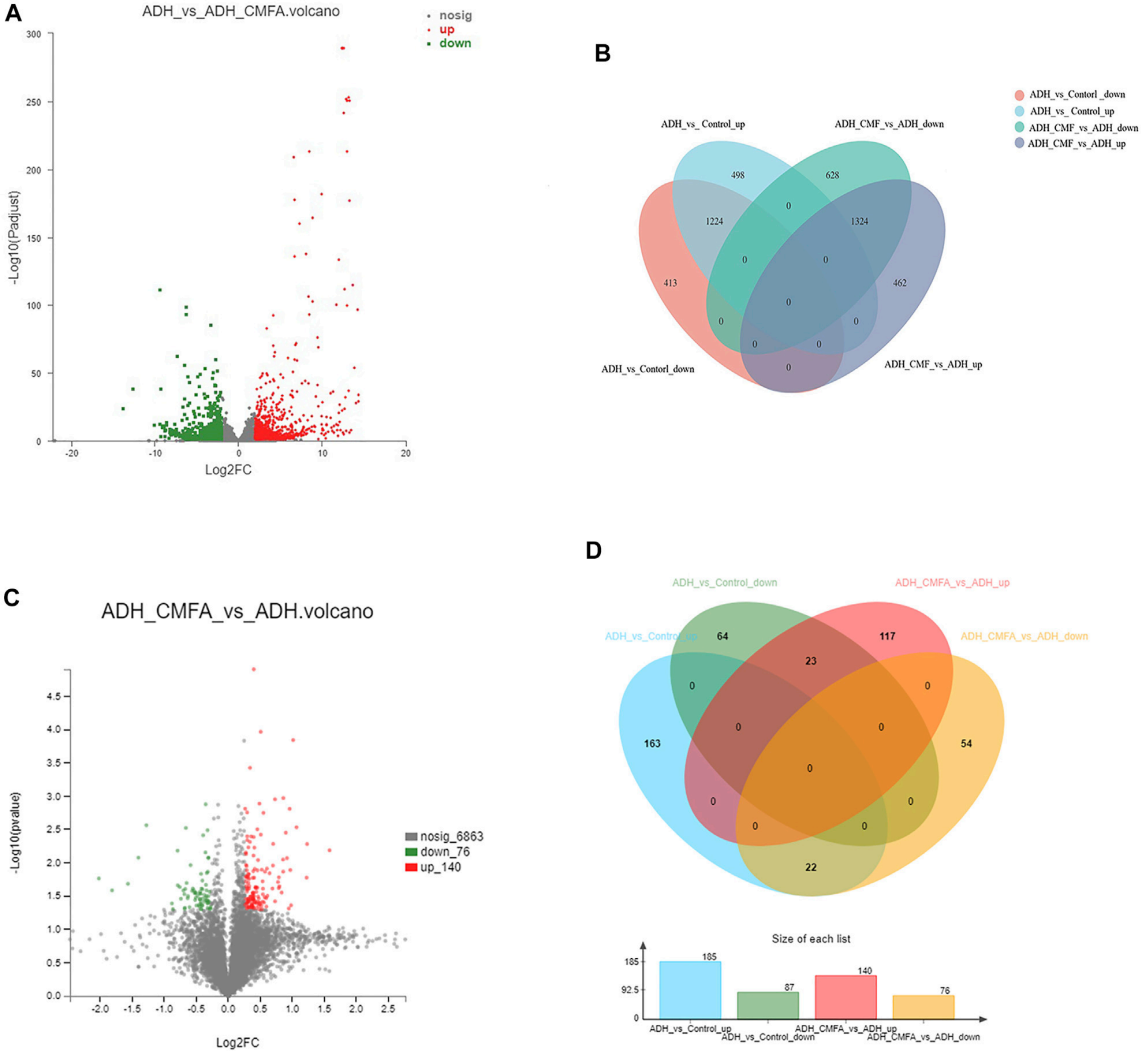
Number of differentially expressed genes and proteins. **(A)** mRNA volcanogram of differential expression **(B)** mRNA venn diagram of differential expression **(C)** proteins volcanogram of differential expression **(D)** proteins venn diagram of differential expression.

In order to understand the multifaceted mechanism of CMF on ADH model, we carried out RNA sequencing analysis to obtain the mRNA expression of tissues from the Control group, ADH group and ADH-CMF group. Totally, 2,548 differential genes were screened by bioinformatics analysis. There were 1,637 genes with low expression in the ADH group, and the CMF treatment increased the expression of 1,224 genes (Supplementary Table S3) compared with the control group (Figures 2A,B). At the same time, 1786 genes were highly expressed in the ADH group compared with the control group, and CMF decreased the expression of 1,324 genes (Supplementary Table S4).

### Proteomic Results

#### Protein Identification

**FIGURE 3:**
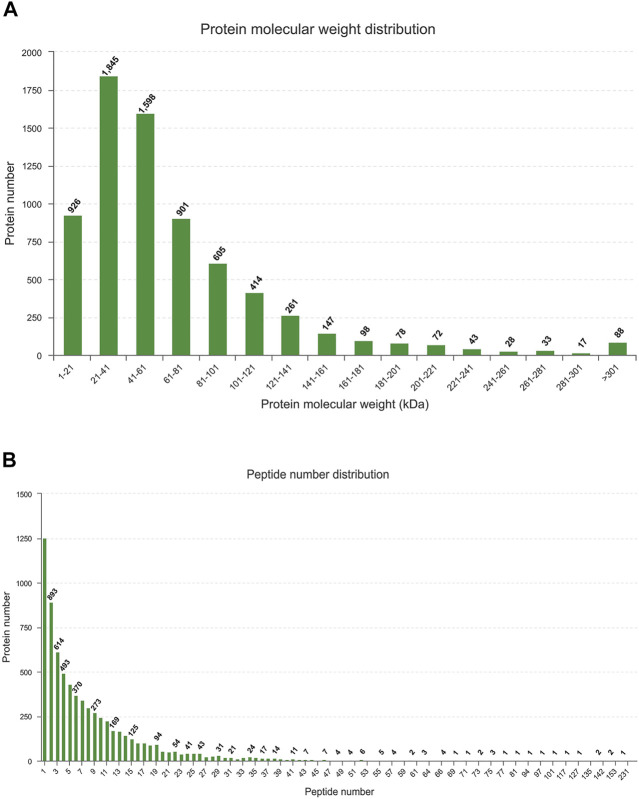
Identification and analysis of proteins **(A)** Distribution of the identified proteins among different molecular-weight classes. **(B)** Distribution of proteins containing different numbers of identified peptides.

The LC-MS/MS generated a total of 60,425 unique peptides, and 21,424 proteins were identified. The number of identified proteins with molecular weight in the range of 1–21 kD, 21–41 kD, 41–61 kD, 61–81 kD, and 81–101 kD was 926 (4.32%), 1,845 (8.61%), 1,598 (7.46%), 901 (4.2%), and 605 (2.82%), respectively, whereas 72.59% of proteins had a molecular weight >101 kD (Figure 1A). Most of the identified proteins (1,225) had unique peptides, and about 73.24% of the identified proteins had three or more peptides (Figure 3).

#### Differential Protein Analysis

In order to explore the potential molecular mechanism of CMF against colorectal adenoma, the proteomic method based on TMT was used to detect the differentially expressed proteins in the Control vs ADH or ADH-CMF group. After combining the data from two and three repetitions of each group, 45 differential proteins were screened. The results showed that 87 proteins were expressed at low level in the ADH group compared with the Control group, and CMF increased the expression of 23 proteins (Supplementary Table S5). At the same time, 185 proteins were overexpressed in the ADH group compared with the Control group, and CMF decreased the expression of 22 proteins (Figures 2C,D) (Supplementary Table S6).

#### Association Analysis of Proteomics and Transcriptomics

**FIGURE 4:**
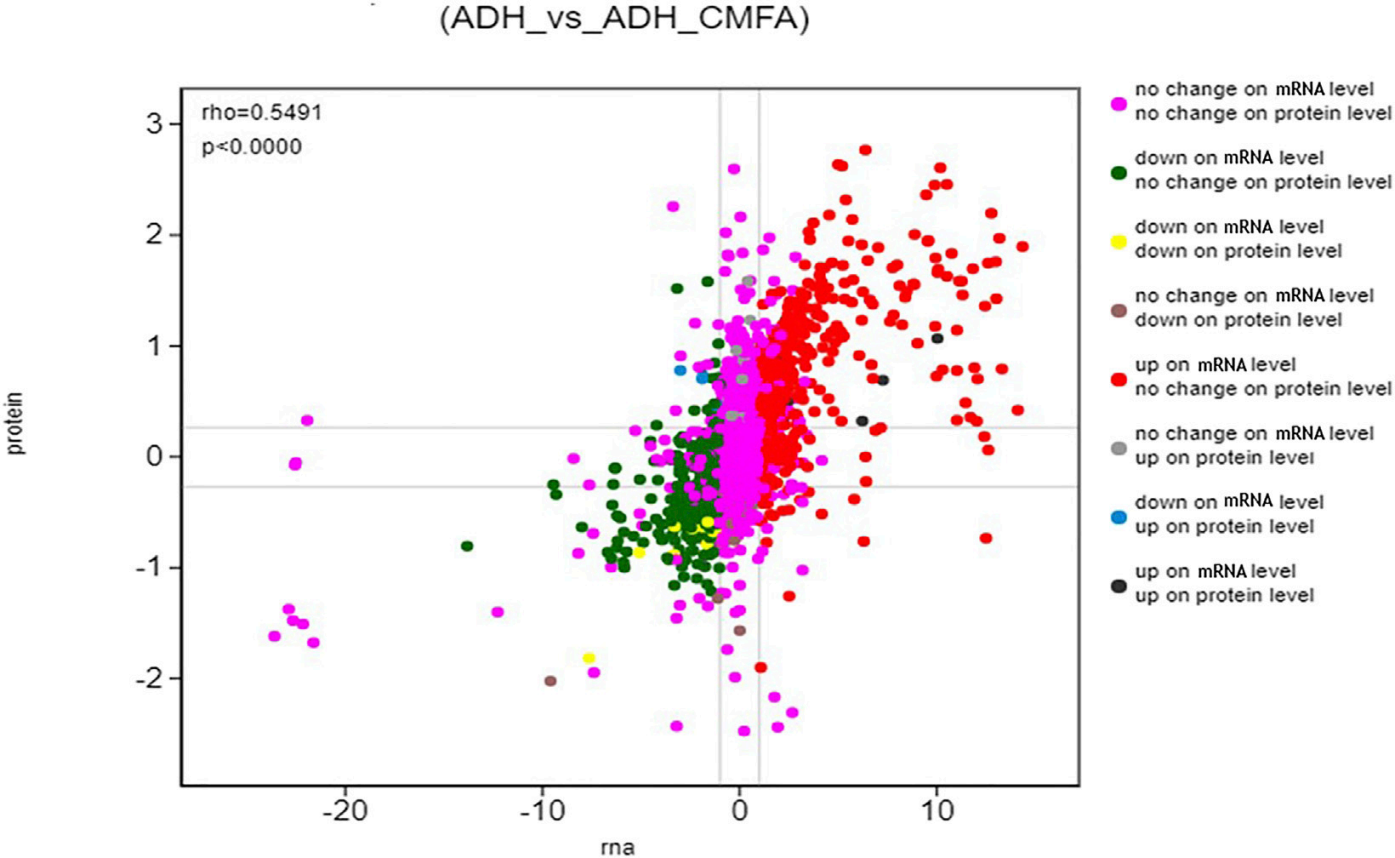
Differential expression analysis (ADH_vs_ADH_CMF) The ordinate represents the expression multiple difference of the protein in a pair of comparison groups, the log2 (ratio of protein). The abscissa represents the expression of multiple difference of the corresponding transcript in the comparison group, the log2 (ratio of gene), logarithm difference takes the logarithmic value respectively; each point represents a protein and its associated transcript, the upper left corner of the picture rho represents the Pearson correlation coefficient between the two groups, *p* represents the correlation test *p* value; rho <0 indicates negative correlation. rho > 0 indicates positive correlation; rho = 0 indicates no correlation.

In this study, the correlation between proteome and transcriptome in the Control-ADH VS ADH-CMF was analyzed based on the expression results of mRNA and protein levels, and the results were shown in Figure 4. The correlation coefficient of differential proteins and genes was 0.5491, presenting a positive correlation. The cross-linking analysis of differential genes and differential proteins showed that nine genes were significantly regulated by CMF not only at the transcriptional level, but also the proteins encoded by these nine genes. Among them, five genes were up-regulated and four genes were down-regulated, as shown in Table 3.

**TABLE 3:** Differential targets obtained by cross-analysis of transcriptome and proteome.

Number	Proteome	Transcriptome	Target	Up or down regulation	KEGG
1	ENSMUSP00000112770.2	ENSMUSG00000084174	Sycn	down	—
2	ENSMUSP00000078088.6	ENSMUSG00000024411	Aqp4	down	Bile secretion, Vasopressin-regulated water reabsorption
3	ENSMUSP00000000642.4	ENSMUSG00000000628	Hk2	down	HIF-1 signaling pathway, carbon metabolism
4	ENSMUSP00000122863.1	ENSMUSG00000030116	Mfap5	down	—
5	ENSMUSP00000029367.5	ENSMUSG00000027792	Bche	Up	Tryptophan metabolism
6	ENSMUSP00000033241.5	ENSMUSG00000030946	Lhpp	Up	oxidative phosphorylation, PI3K-Akt signaling pathway
7	ENSMUSP00000066927.3	ENSMUSG00000021944	Gata4	Up	cGMP-PKG signal pathway, cell senescence
8	ENSMUSP00000134082.1	ENSMUSG00000035561	Aldh1b1	Up	glycolysis, fatty acid metabolism
9	ENSMUSP00000112472.1	ENSMUSG00000032572	Col6a4	Up	PI3K-Akt signaling pathway

### GO and KEGG Enrichment Analysis

**FIGURE 5:**
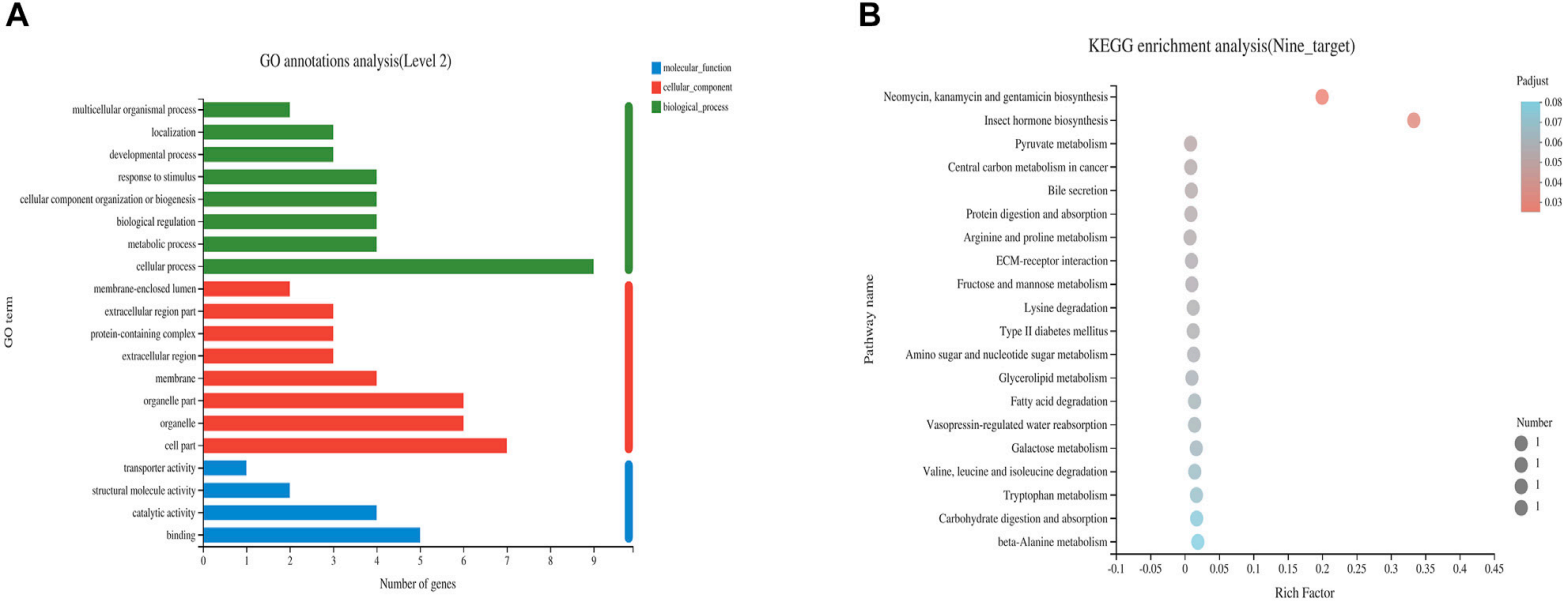
Enrichment analysis of GO **(A)** and KEGG **(B)** of differential genes after CMF intervention.

Nine differentially expressed targets were enriched and analyzed in GO and KEGG pathways, and the enrichment was found in three categories: biological processes, molecular functions and cellular components. The KEGG pathways were obtained under the first-level classification of GO. GO enrichment results showed that the differentially expressed targets were significantly enriched in cellular components such as membrane, protein-containing complex and membrane-enclosed lumen, molecular functions such as catalytic activity, transporter activity and binding, and significant enrichment in biological processes such as metabolic process, developmental process and biological regulation (Figure 5A). The enrichment results of KEGG pathway showed that the differential expression targets were mainly involved in carbon metabolism, tryptophan metabolism, oxidative phosphorylation, cGMP-PKG signal pathway, cell senescence, glycolysis, fatty acid metabolism, PI3K-Akt signaling pathway and other signaling pathways (Figure 5B).

### Verification

**FIGURE 6:**
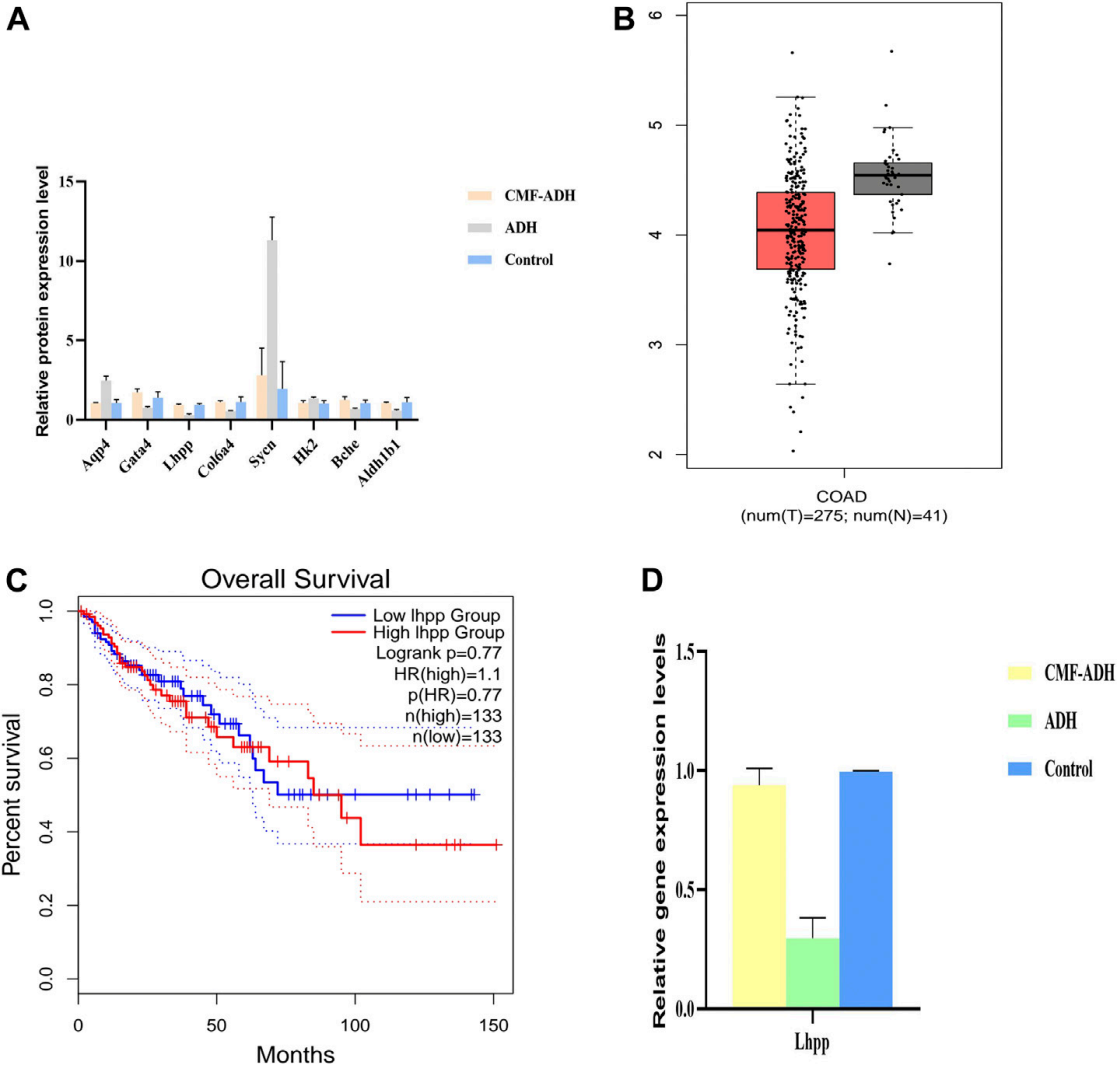
Validation of the Transcriptome and proteome data in intestinal tissues **(A)** DEPs were selected and validated by PRM. Each data point is calculated from the average of biological triplicates. Results of the PRM analysis were consistent with TMT data. **(B)** Decreased expression of LHPP in colorectal cancer tissues when compared with normal tissues from the TCGA database **(C)** No significant difference in overall survival was observed between patients from the TCGA database with high LHPP expression. ****p < 0.0001 **(D)**. Comparison of relative gene expression level of the selected differentially regulated proteins measured by qPCR. Each data point is calculated from the average of biological triplicates.

The relative contents of Sycn, Aqp4, Hk2, Mfap5, Bche, Gata4, Aldh1b1, Col6a4 and Lhpp proteins in the intestinal tissue samples were detected by PRM method. The results showed that, compared with the control group, the expressions of Aqp4, Sycn and Hk2 in the model group were up-regulated, while the expressions of Lhpp, Gata4, Col6a4, Bche and Aldhlb1 were down-regulated. The expression was reversed in the CMF treatment group (Figure 6A).

In order to determine the expression of target proteins in CRA, we analyzed the expression of Sycn, Aqp4, Hk2, Mfap5, Bche, Gata4, Aldh1b1, Col6a4 and Lhpp in GEPIA database (http://gepia.cancer-pku.cn/). Referring to relevant studies, Lhpp was selected as the core target, and its expression was consistent with that in the database. Lhpp expression was markedly lower in CRC tissues compared with that in the normal tissues (Figure 6B). However, no significant difference in the overall survival was observed between patients with high and low expression of LHPP (Figure 6C).

The relative content of Lhpp mRNA in intestinal tissue samples was detected by qPCR. The results showed that the expression of Lhpp mRNA in the model group was down-regulated compared with the control group. The expression in the treatment group was increased and similar to the control group (Figure 6D), and the difference was statistically significant (*p* < 0.05).

### Effects of CMF on Intestinal Microflora Structure Operational Taxonomic Unit cluster Analysis

**FIGURE 7:**
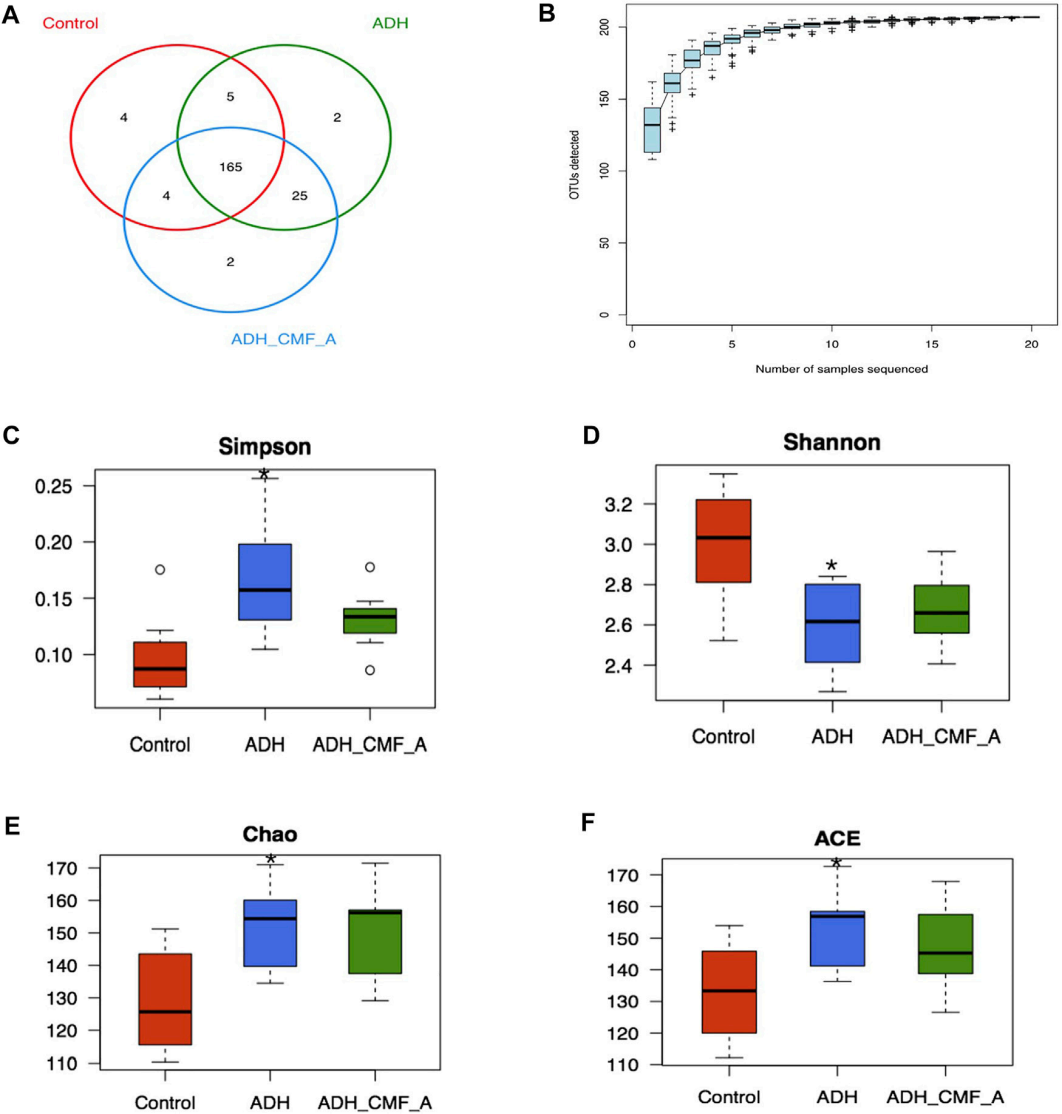
Effects of CMF on the structure and diversity of intestinal microflora **(A)** Venn diagram of OTU distribution differences between groups; **(B)** Species accumulation curve; **(C)** Comparison box of Simpson index for diversity; **(D)** Comparison box of diversity Shannon index; **(E)** Box chart of diversity Chao index comparison; **(F)** Box plot of diversity ACE index comparison; Compared with Control mice, *p < 0.05.

All OTUs were divided, and 97% of OTUs at similar levels were statistically analyzed for biological information. R language was used to calculate the number of OTU shared by each group of samples, and the Venn diagram of sample information was obtained (Figure 7A). There are 207 OTUs in the three groups, including 178 OTUs in the Control group, 197 OTUs in the ADH group, 196 OTUs in the ADH-CMFA group, 4 OTUs in the Control group, two outs in the ADH group and 2 OTUs in the ADH-CMFA group. The difference of OTU distribution between groups in Venn diagram showed the area of microflora overlap, which directly reflects the similarity of OTU composition among groups.

### Mice Intestinal Microbial Species Accumulation Curves (Specaccum Curve) Analysis

On the basis of 97% similarity OTU, the R software statistics was used to further obtain a species accumulated SPECCUM curve, as shown in Figure 7B. The results showed that as the expansion of the measurement volume increases, the curve is initially presented as a sharp rise, indicating that there is a large number of species in the community, and it is necessary to increase the sample detection. As the number of samples increases, the accumulation curve tends to be gentle, indicating that the intestinal flora detection is sufficient and can be analyzed.

### Analysis of Alpha Diversity of Intestinal Microbial Species

Alpha diversity analyzes the abundance and diversity of the reactive intestinal strain, CHAO and ACE can calculate the strife abundance, the greater the value, the more the flora; Shannon and SIMPSON can calculate the flora diversity, of which the larger the Shannon value, the more the flora diversity. The SIMPSON index is inversely proportional to the flora. As shown in Figures 7C–F, the SIMPSON index is significantly reduced, and the Shannon index is significantly reduced, and the ACE index is significantly increased, and has statistical significance in the ADH-CMF group compared with the model group (*p* < 0.05). With the CMF effective component intervention, the SIMPSON index decreased, the Shannon index increased slightly, the CHAO and ACE index also reduced in the ADH-CMF group compared with the model group. The variation of the above index is not obvious with no statistical difference, but the change trend is consistent with the trend of normal group mice.

### Effects of CMF on the Important Intestinal Flora of Mice at Genus Level

**FIGURE 8:**
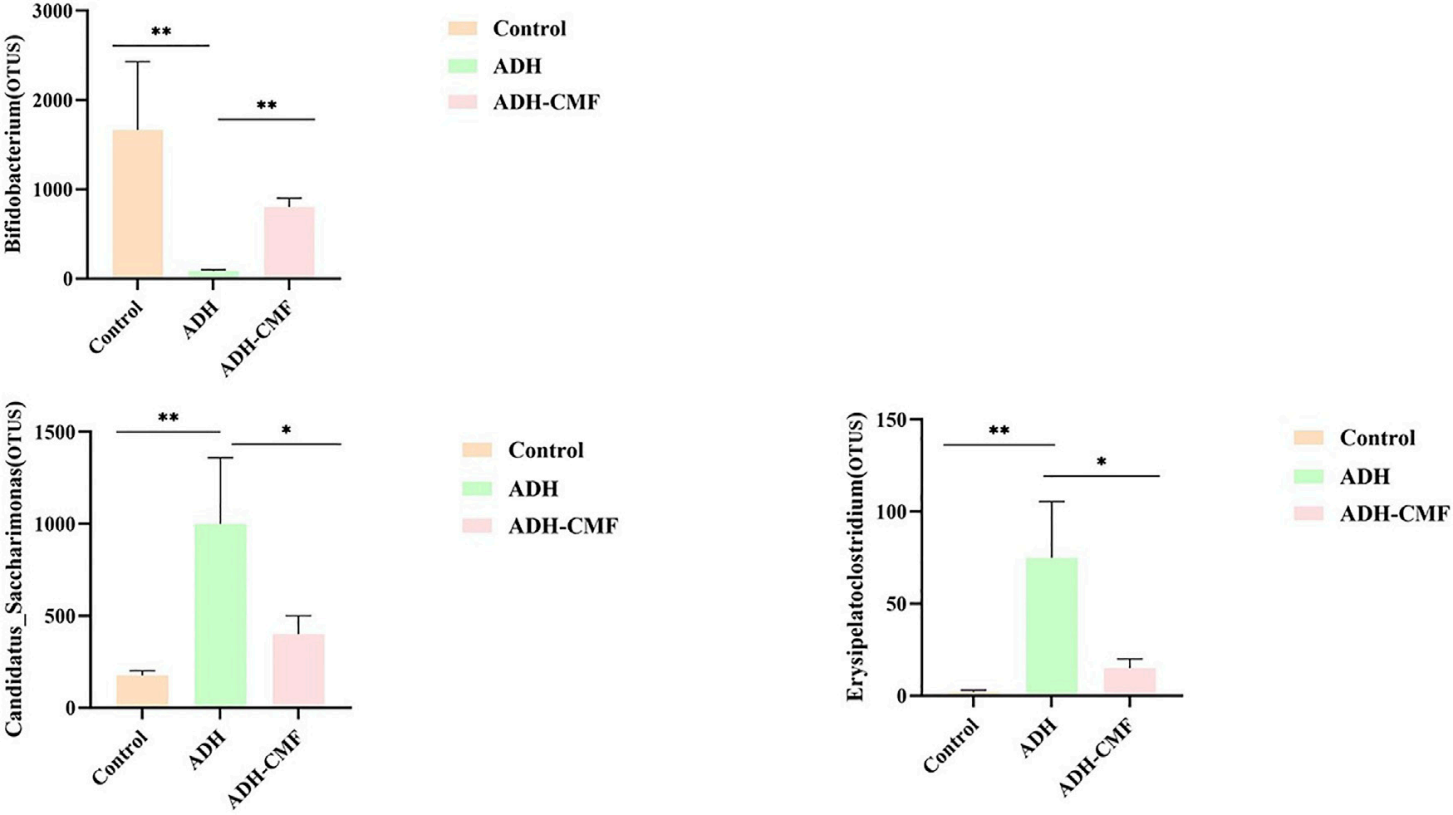
The differential analysis of intestinal flora at the genus level **p < 0.001; *p < 0.01.

The statistical analysis was conducted on the intestinal flora at the genus level in the three groups of mice. The changes were consistent with the control group after the intervention with CMF, as shown in Figure 8. The abundance of Bifidobacterium in the control group was high, but the abundance of Bifidobacterium in the model group was reduced. After the intervention with CMF, the abundance of Bifidobacterium in the intestinal tract increased significantly. In contrast, the abundance of intestinal flora Candidatus_Saccharimonas and Erysipelatoclostridium, that were abundant in the model mice, was significantly reduced after the intervention with CMF. The change of the above three intestinal microflora contents was consistent with that of normal mice, and there was a statistical significance.

## Discussion

As one of the key process in the pathogenesis of colorectal cancer, CRA has the characteristics of two-way transformation, which is a focus of research for the prevention of colorectal cancer (Corley et al., 2014). It is of great significance to explore the mechanism of the occurrence of carcinogenesis of CRA, and to prevent or reverse its occurrence and development (Dubé et al., 2017). In recent years, sequencing technology and bioinformatics analysis technology have rapidly developed (Shen et al., 2019). Transcriptomics and proteomics have become tools for research, that have unique advantages to study the mechanisms of diseases (Hwang et al., 2018). The combination of proteomics and transcriptomics can screen core targets from massive databases more effectively than single omic analysis (Chalmel and Rolland, 2015; Li L. et al., 2021). According to the change of size and the number of adenomas, the length of colon and the pathological condition of intestinal mucosal hyperplasia, CMF has been proved to be an effective drug for the treatment of CRA. This study explored the potential mechanism of CMF in the treatment of CRA.

The differential genes and proteins of colorectal adenoma treated with or without CMF were screened and validated by combining RNA-seq transcriptome sequencing technology and high-throughput proteomic quantification technology with TMT markers. The comprehensive and dynamic changes of gene and protein content in intestinal tissue of CRA mice under the action of CMF were determined. Through analysis of the data of the control, ADH and ADH-CMF groups, 2,548 overlapping genes and 45 overlapping proteins were identified among the two combinations of differentially expressed genes. Sycn, Aqp4, Hk2, Mfap5, Bche, Gata4, Aldh1b1, Col6a4 and Lhpp are regulated by CMF not only at the transcriptional level, but also in the proteins encoded by these nine genes. GO analysis showed that these targets were mainly involved in the biological processes such as cellular process, metabolic process and biological regulation. KEGG analysis identified the involvement of oxidative phosphorylation, cell senescence, and metabolic pathways. It shows that the treatment of CRA by CMF is multi-target.

CRA has a high degree of heterogeneity and genomic instability, while the same gene has different regulatory effects on different tumors. Based on the GEPIA database, Lhpp was selected as the core target. The results of PRM and qPCR were consistent with the results of RNA-Seq transcriptome sequencing and TMT analysis. CMF can effectively regulate the expression of the gene, which is consistent with the pathogenesis of the disease. Lhpp is a histidine phosphatase, opposite to histidine kinase, which can reverse the phosphate group connected to histidine on proteins (Hindupur et al., 2018). A study showed that Lhpp mutation and expression decreased in esophageal, head and neck, stomach, bladder, breast, skin, liver, lung and pancreatic tumors, indicating that the increase of histidine phosphorylation is related to tumorigenesis (Li et al., 2019; Li C. et al., 2021). A recent study revealed that dysregulation of Lhpp was frequently observed in CRC tissues and was positively correlated with tumor severity and poor prognosis. Overexpression of Lhpp impeded CRC cell growth and proliferation *in vitro*, and was associated with a change in p53 expression and PI3K/AKT activity (Hou et al., 2020). In contrast, silencing of Lhpp significantly promoted cell growth and proliferation by modulating the PI3K/AKT signaling pathway. The phosphatidylinositol-3-kinase/protein kinase B (PI3K/AKT) signaling pathway is one of the most classical pathways involved in tumorigenesis (Xie et al., 2019). Previous results indicated that cell cycle was arrested in the G0/G1 phase after improved expression of Lhpp, while reduction of Lhpp displayed the opposite results (Li Z. et al., 2021). Therefore, the role of Lhpp is similar to that of tumor suppressor gene, indicating that CMF may inhibit the occurrence of CRA by regulating the expression of Lhpp. Lhpp is expected to become one of the effective serological markers for the diagnosis of CRA and a potential therapeutic target.

Previous studies have shown that intestinal flora is related to CRA formation, and proper regulation of intestinal flora can improve the intestinal environment and inhibit adenoma formation. Bifidobacteriaceae are probiotics in the intestine and play an important role in reducing inflammatory response, reducing intestinal permeability, and maintaining integrity of intestinal epithelium (Lugli et al., 2017). Some studies have shown that Bifidobacteriaceae can produce acetate to prevent intestinal infection (Fukuda et al., 2011; Zheng et al., 2019). In this study, the abundance of the intestinal Bifidobacteriaceae in the ADH mouse was significantly reduced. After intervention by CMF, the Bifidobacteriaceae content increased. The change of intestinal flora, particularly the reduction of the Candidatus_saccharimonas bacterial content, was found to cause intestinal barrier failure in the acute necrotizing pancreatitis mouse model (Chen et al., 2017). There is also a study showed that Candidatus_saccharimonas was related to the use of nitrogen. In our experiment, the Candidatus_ saccharimonas in colorectal adenoma mice was found to be higher than that of the control group. After the intervention by CMF, the content was significantly reduced, and it was speculated that Candidatus_ saccharimonas is a pathogens in the progress of intestinal adenoma. Studies have shown that the treatment of xylan butyrate affects the abundance of erysipelatoclostridium flora in the DSS induced ulcerative colitis model of C57BL/6 mice (Zha et al., 2020). This study found that the content of erysipelatoclostridium in ADH mice increased significantly, and CMF could significantly reduce the content of erysipelatoclostridium in the intestine, so it is speculated that Erysipelatoclostridium flora can be a potential pathogenic bacteriain the development of intestinal adenoma. Previous studies showed that Candidatus_Saccharimonas was negatively correlated with LHPP (Guo et al., 2021), and CMF was speculated to play a major role in the anti-CRA effect of Candidatus_Saccharimonas and LHPP, but the relationship between the two is not clear. Further research will be carried out by combining metagenomic sequencing and metabonomics. Therefore, the intervention of CMF regulated the structure of intestinal flora, increased the content of probiotics and reduced the abundance of pathogenic bacteria in intestinal tract.

In conclusion, CMF regulates the expression of key target LHPP through oxidative phosphorylation, PI3K/Akt and other signaling pathways, thus inhibiting the proliferation of intestinal adenoma cells and inducing apoptosis. Moreover, CMF regulates the structure and abundance of intestinal flora and reverses the disorder of intestinal flora, and therefore achieves the purpose of anti-CRA.

## Data Availability

The sequencing data presented in the study are deposited in the SRA repository, accession number PRJNA777288; The mass spectrometry proteomics data have been deposited to the ProteomeXchange Consortium via the PRIDE partner repository with the dataset identifier PXD029296.
