# Supplementary material for: Exploring the Mechanism of Action of Canmei Formula Against Colorectal Adenoma Through Multi-Omics Technique
Source: Front Cell Dev Biol. 2021 Nov 29;9:778826. doi: 10.3389/fcell.2021.778826 (PMC8672438; doi:10.3389/fcell.2021.778826)
Supplement: Supplementary file 2 [file Table1.docx]

**S Table1 Major chemical constituents identified in CMF.**

| No. | Name | RT (min) | Formula | Molecular weight | Area (max.) |
| --- | --- | --- | --- | --- | --- |
| 1 | DL-Arginine | 1.28 | C6 H14 N4 O2 | 174.1117 | 1300061.86 |
| 2 | L(-)-Carnitine | 1.37 | C7 H15 N O3 | 161.10527 | 9750518.42 |
| 3 | DL-Stachydrine | 1.40 | C7 H13 N O2 | 143.09471 | 1508022.19 |
| 4 | Adenine | 1.42 | C5 H5 N5 | 135.05457 | 12469462.57 |
| 5 | Guanine | 1.43 | C5 H5 N5 O | 151.04946 | 7435505.89 |
| 6 | Hypoxanthine | 1.57 | C5 H4 N4 O | 136.03858 | 12616671.18 |
| 7 | Nicotinic acid | 1.58 | C6 H5 N O2 | 123.03221 | 1086991.17 |
| 8 | L-Tyrosine | 1.64 | C9 H11 N O3 | 181.07401 | 6502680.25 |
| 9 | Adenosine | 1.69 | C10 H13 N5 O4 | 267.09676 | 26955456.82 |
| 10 | Adenine | 1.69 | C5 H5 N5 | 135.05457 | 3750819.71 |
| 11 | 2-Hydroxycinnamic acid | 1.72 | C9 H8 O3 | 164.04745 | 37877896.83 |
| 12 | Hypoxanthine | 1.76 | C5 H4 N4 O | 136.03858 | 2774356.88 |
| 13 | L-Norleucine | 2.07 | C6 H13 N O2 | 131.09474 | 55189905.01 |
| 14 | Alanyltyrosine | 2.81 | C12 H16 N2 O4 | 230.12691 | 433071.84 |
| 15 | Nornicotine | 2.88 | C9 H12 N2 | 148.10023 | 477190.87 |
| 16 | 2-Oxindole | 3.52 | C8 H7 N O | 151.06347 | 420066.93 |
| 17 | 2^′^-Deoxyadenosine | 3.54 | C10 H13 N5 O3 | 251.10184 | 148660.32 |
| 18 | Glycyl-L-leucine | 4.17 | C8 H16 N2 O3 | 188.11627 | 4092286.76 |
| 19 | Chlorogenic acid | 4.80 | C16 H18 O9 | 354.09487 | 36388021.14 |
| 20 | tert-Butyl N-[1-(aminocarbonyl)-3-methylbutyl]carbamate | 5.41 | C11 H22 N2 O3 | 230.16338 | 2831504.35 |
| 21 | D-(+)-Tryptophan | 5.45 | C11 H12 N2 O2 | 204.08996 | 21112273.16 |
| 22 | 3,4-Dihydroxybenzaldehyde | 5.90 | C7 H6 O3 | 138.03188 | 196915.08 |
| 23 | Leucylproline | 5.94 | C11 H20 N2 O3 | 228.14765 | 2951420.35 |
| 24 | 7-Hydroxycoumarine | 6.51 | C9 H6 O3 | 162.03159 | 3160911.99 |
| 25 | 6-Methylquinoline | 6.93 | C10 H9 N | 143.07372 | 1669486.73 |
| 26 | 3-Phenylpropanoic acid | 7.18 | C9 H10 O2 | 150.06808 | 50678.13 |
| 27 | 3-O-feruloyl-D-quinic acid | 7.63 | C17 H20 O9 | 368.11073 | 285397.82 |
| 28 | 4-Coumaric acid | 7.90 | C9 H8 O3 | 164.04747 | 616864.76 |
| 29 | Rutin | 7.92 | C27 H30 O16 | 610.15385 | 383860.64 |
| 30 | N-Acetyl-L-leucine | 7.95 | C8 H15 N O3 | 173.10535 | 172744.27 |
| 31 | Quercetin-3β-D-glucoside | 8.13 | C21 H20 O12 | 464.09641 | 532784.34 |
| 32 | 3,5-Dimethoxybenzoic acid | 8.22 | C9 H10 O4 | 182.05817 | 712514.01 |
| 33 | Quercetin | 8.52 | C15 H10 O7 | 302.0428 | 150731.57 |
| 34 | Kaempferol-7-O-glucoside | 8.53 | C21 H20 O11 | 448.10099 | 407020.68 |
| 35 | (3R,4S)-4,6,8-Trihydroxy-7-methoxy-3-methyl-3,4-dihydro-1H-isochromen-1-one | 8.89 | C11 H12 O6 | 222.05297 | 117315.38 |
| 36 | (2E)-3-(3,4-Dimethoxyphenyl)acrylic acid | 9.07 | C11 H12 O4 | 208.07356 | 2293865.83 |
| 37 | Ambrosic acid | 10.00 | C15 H20 O4 | 264.13609 | 678788.38 |
| 38 | Andrographolide | 10.34 | C20 H30 O5 | 350.20662 | 1261013.91 |
| 39 | Ipratropium | 11.70 | C20 H29 N O3 | 331.21202 | 224430.08 |
| 40 | Pinolenic acid | 13.35 | C18 H30 O2 | 278.22456 | 293978.53 |
| 41 | α-Eleostearic acid | 14.23 | C18 H30 O2 | 278.22457 | 1744840.46 |

**S Table 2 Sequencing data of cDNA library of each sample**

| Sample | Clean reads | Clean bases | Error rate(%) | Q20(%) | Q30(%) | GC content(%) |
| --- | --- | --- | --- | --- | --- | --- |
| ADH_1 | 50671288 | 7.58E+09 | 0.0245 | 98.22 | 94.7 | 50.05 |
| ADH_2 | 52078576 | 7.78E+09 | 0.0248 | 98.08 | 94.4 | 51.02 |
| ADH_3 | 55707774 | 8.32E+09 | 0.0245 | 98.21 | 94.69 | 50.2 |
| ADH_CMFA_1 | 50016160 | 7.48E+09 | 0.0241 | 98.35 | 95.04 | 51.35 |
| ADH_CMFA_2 | 59653478 | 8.91E+09 | 0.0242 | 98.32 | 94.95 | 51.24 |
| ADH_CMFA_3 | 51599718 | 7.72E+09 | 0.0243 | 98.29 | 94.87 | 51.28 |
| Control_1 | 54625726 | 8.16E+09 | 0.0238 | 98.48 | 95.34 | 50.91 |
| Control_2 | 52402872 | 7.84E+09 | 0.0241 | 98.37 | 95.08 | 51.11 |
| Control_3 | 48445552 | 7.25E+09 | 0.0242 | 98.34 | 94.96 | 50.87 |
