# Supplementary figures and images for: Exploring the Mechanism of Action of Canmei Formula Against Colorectal Adenoma Through Multi-Omics Technique
Source: Front Cell Dev Biol. 2021 Nov 29;9:778826. doi: 10.3389/fcell.2021.778826 (PMC8672438; doi:10.3389/fcell.2021.778826)

ESI+


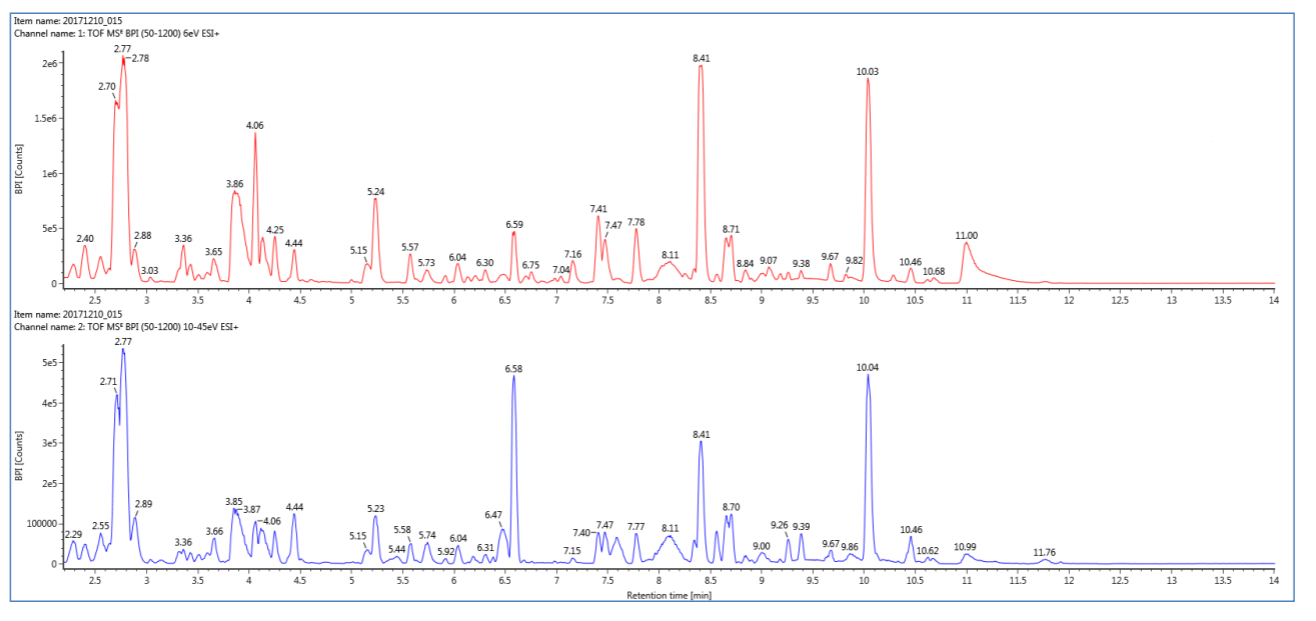


ESI-


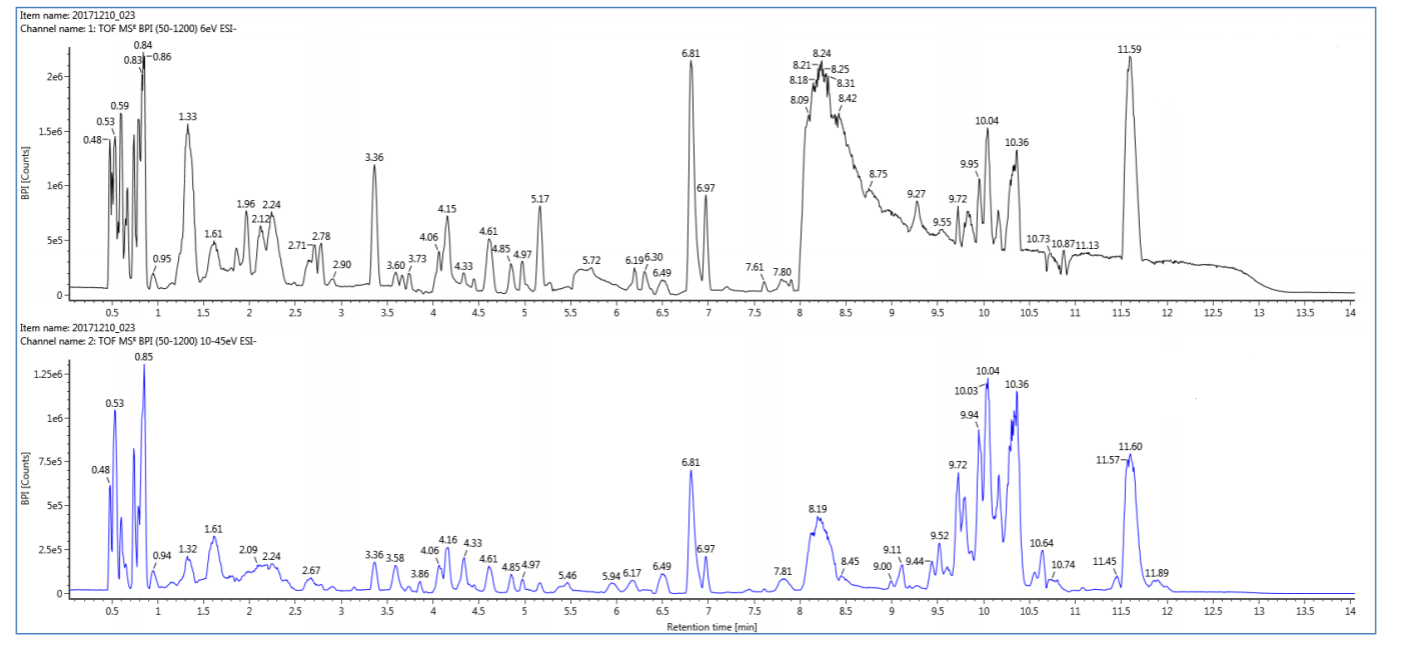


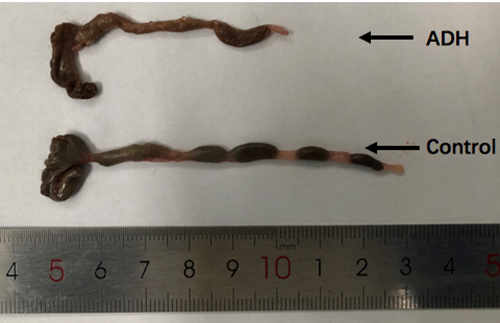


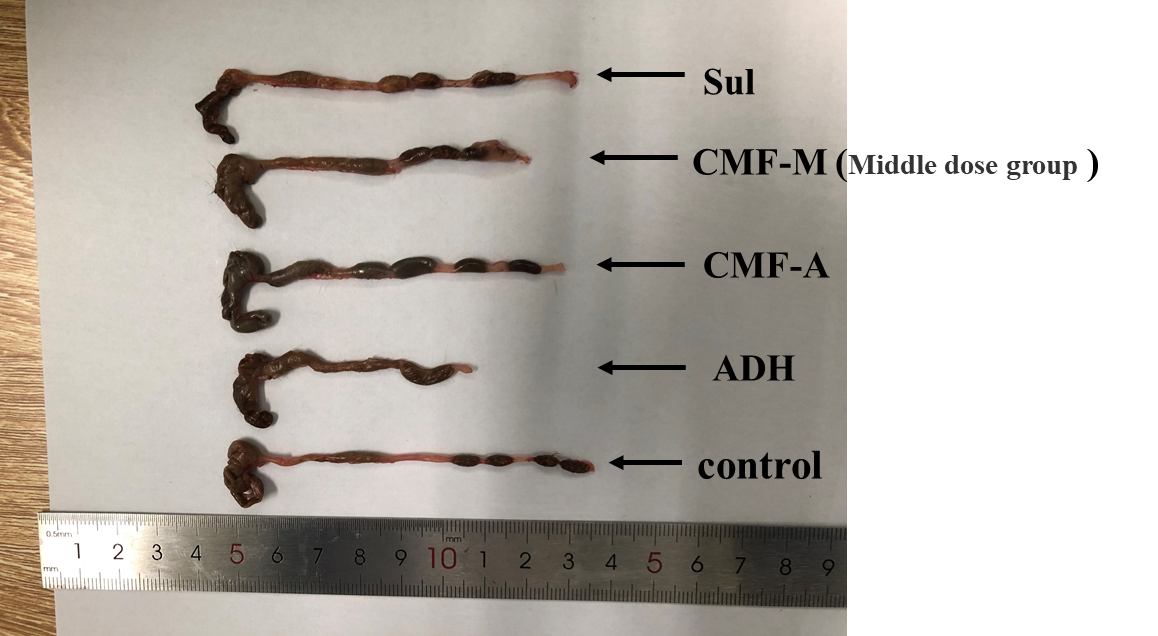


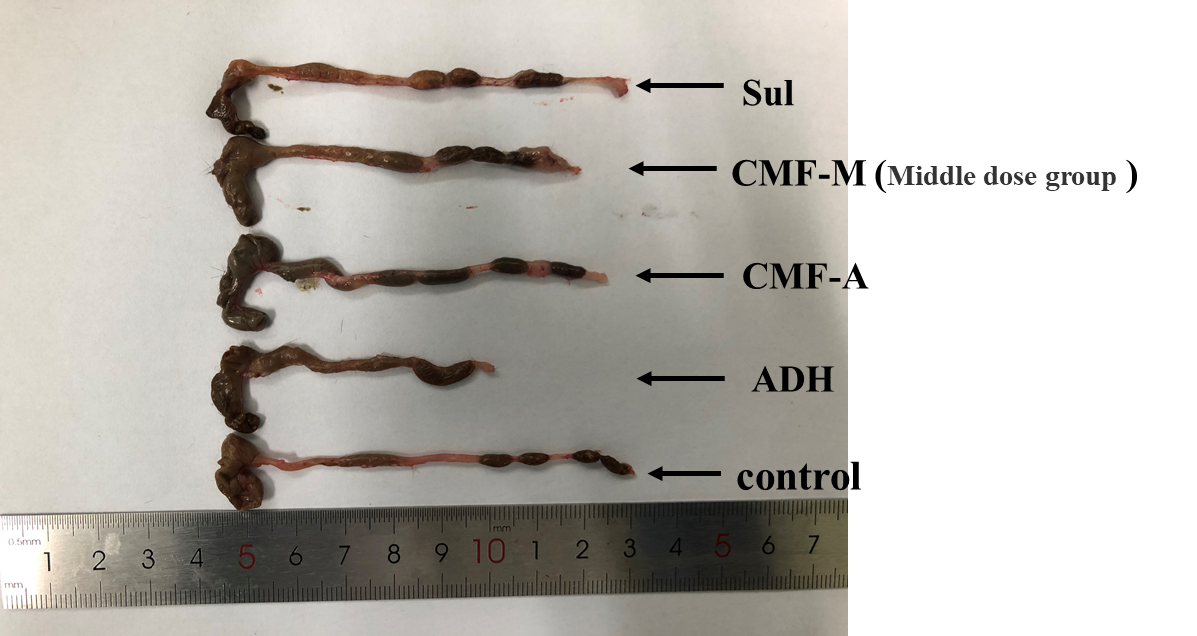


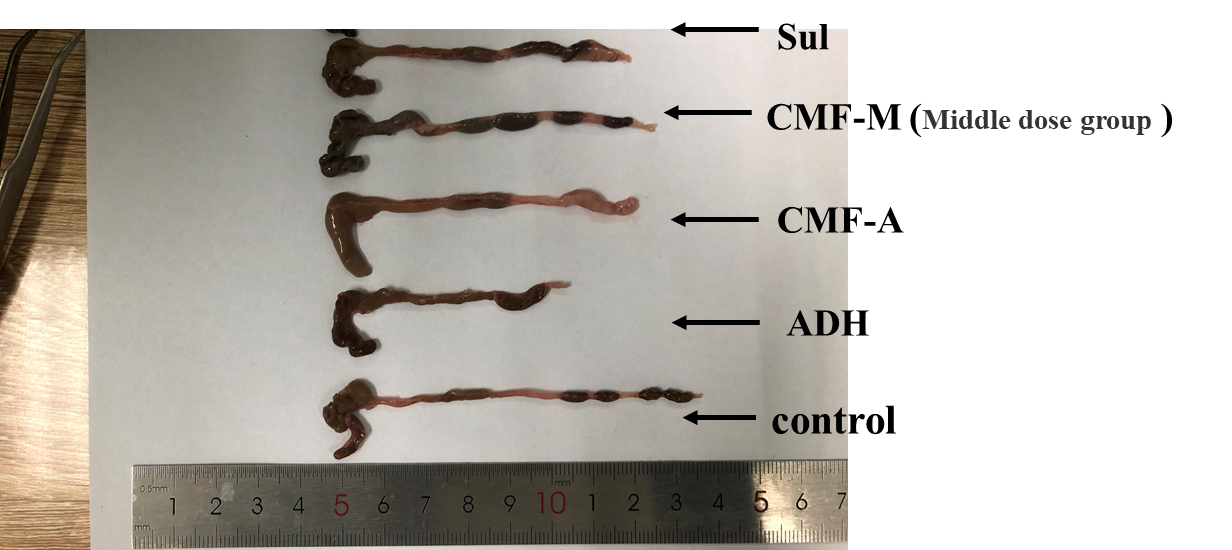

Supplement: Supplementary file 5 [file Table6.docx]
